# Aglianico Grape Pomace Extract Reduces Cardiac Pacemaker Activity by Decreasing Hyperpolarization-Activated Current Density Independently of cAMP Signaling

**DOI:** 10.3390/life16050786

**Published:** 2026-05-08

**Authors:** Roberta De Zio, Maira Certini, Eugenia Pignataro, Daniela Russo, Simona Ida Scorza, Serena Milano, Giuseppe Procino, René Massimiliano Marsano, Maria Svelto, Isabella Maiellaro, Luigi Milella, Monica Carmosino, Andrea Gerbino

**Affiliations:** 1 Department of Biosciences, Biotechnology and Environment, University of Bari Aldo Moro, 70125 Bari, Italy; maira.certini@uniba.it (M.C.); eugenia.pignataro@uniba.it (E.P.); simona.scorza@uniba.it (S.I.S.); giuseppe.procino@uniba.it (G.P.); renemassimiliano.marsano@uniba.it (R.M.M.); maria.svelto@uniba.it (M.S.); 2 Department of Health Sciences, University of Basilicata, 85100 Potenza, Italy; daniela.russo@unibas.it (D.R.); serena.milano@unibas.it (S.M.); luigi.milella@unibas.it (L.M.); monica.carmosino@unibas.it (M.C.); 3 School of Life Sciences, Department of Neuroscience, University of Nottingham, Nottingham NG7 2RD, UK; isabella.maiellaro@nottingham.ac.uk

**Keywords:** grape pomace extract, funny current, HCN channels, cardiac pacemaker, diastolic depolarization, polyphenols, *Drosophila melanogaster*, chronotropy, cAMP, HL-1 cardiomyocytes

## Abstract

Grape pomace extract (GPE) from *Vitis vinifera* L. cv. Aglianico is rich in polyphenols with recognized cardioprotective properties, yet its direct electrophysiological effects on spontaneous cardiac activity have not been previously investigated. Here, we examined the chronotropic effects of GPE using two complementary models: HL-1 cardiomyocytes, assessed by whole-cell patch-clamp and intracellular Ca^2+^ imaging, and the *Drosophila melanogaster* larval heart tube, evaluated by optical recording. In HL-1 cells, chronic treatment with 25 µg/mL GPE for 48 h significantly reduced potential spontaneous action frequency and selectively prolonged the diastolic depolarization phase without altering action potential morphology, depolarization-activated currents, or cytosolic Ca^2+^ homeostasis. GPE reduced the hyperpolarization-activated funny current (I_f_) density without shifting its voltage dependence. GPE-treated cells retained cAMP sensitivity, as both isoproterenol and intracellular 8-Br-cAMP significantly increased I_f_ amplitude, while ELISA quantification confirmed that global cAMP levels were unaffected by GPE. In *Drosophila* larvae, a cAMP-independent myogenic preparation, GPE administered in the diet significantly reduced heart rate. These findings demonstrate that Aglianico GPE exerts a negative chronotropic effect through a mechanism that reduces functional I_f_ density without altering cAMP availability or HCN channel voltage dependence, and reveal a cAMP-independent component of action conserved across phylogenetically distant species.

## 1. Introduction

The sinoatrial node is the primary pacemaker of the mammalian heart, generating spontaneous rhythmic action potentials that drive each heartbeat. A defining electrophysiological feature of sinoatrial node myocytes is the presence of a slow diastolic depolarization, which gradually brings the membrane potential from the maximum diastolic potential to the threshold for the next action potential. Among the ionic currents that contribute to diastolic depolarization, the cyclic nucleotide-gated (HCN) channels play a central role. HCN channels are hyperpolarization-activated and give rise to the so-called “funny” current (I_f_), named for its unusual property of being activated by membrane hyperpolarization, unlike most inward currents. The I_f_ current is an inward current activated by membrane hyperpolarization and positively modulated by intracellular cAMP; within the voltage range of diastolic depolarization, its degree of activation determines the slope of depolarization and hence the frequency of action potential firing [1]. Four isoforms of HCN channels (HCN1–4) have been identified as the molecular correlates of I_f_. HCN4 is the predominant isoform expressed in the sinoatrial node, and its characteristic properties, slow activation kinetics and strong sensitivity to cAMP closely match those of native I_f_ recorded in sinoatrial node pacemaker cells [2].

The modulation of I_f_ by intracellular cAMP is the primary mechanism through which the autonomic nervous system regulates heart rate. At the molecular level, cAMP binds directly to the cyclic nucleotide-binding domain (CNBD) located at the C-terminus of the HCN channel, inducing an allosteric conformational change that shifts the voltage dependence of channel opening toward more depolarized potentials [3,4]. This shift translates into increased channel activation at physiological diastolic voltages, a steeper diastolic depolarization, and ultimately a higher firing rate. Conversely, reduced cAMP binding produces a hyperpolarizing shift in the activation curve, slowing the diastolic depolarization and decreasing heart rate, the mechanism underlying muscarinic slowing of pacemaking. The CNBD of HCN4 is therefore a critical regulatory node at the convergence of sympathetic and parasympathetic inputs, and its pharmacological manipulation represents an effective and selective means of controlling sinoatrial node rate.

Although the fundamental role of I_f_ in cardiac pacemaking has been well established, interest in natural compounds as modulators of this current has grown considerably in recent years [5], not only for potential therapeutic implications in conditions such as sinus node dysfunction, for which effective long-term pharmacological options remain limited [6], but also as pharmacological tools to dissect the molecular mechanisms of HCN channel regulation. Several natural compounds have been shown to influence I_f_ and sinoatrial node automaticity through distinct mechanisms. Berberine [7] and acehytisine [8] both inhibit hHCN4 currents and slow pacemaker firing in rabbit sinoatrial node preparations, with mechanistic features compatible with channel block. Ginkgo biloba extract and its main constituent bilobalide reduce the slope of diastolic depolarization in rat sinoatrial node cells and inhibit hHCN2- and hHCN4-mediated currents in heterologous expression systems [9]. Most relevant to the present study, Tongmai Yangxin (TMYX), a Traditional Chinese Medicine complex formulation, elicits a reversible, dose-dependent slowing of spontaneous firing in rabbit sinoatrial node cells through a negative shift in the If activation curve by directly antagonizing the cAMP-induced allosteric modulation of HCN channels without requiring changes in cytoplasmic cAMP levels [10]. This mechanism confers on TMYX a pharmacologically distinct mode of action, ensuring rate slowing under basal conditions while preserving the chronotropic reserve during sympathetic stimulation.

In the present study, we investigated the electrophysiological effects of a grape pomace extract (GPE) obtained from the *Vitis vinifera* cv. Aglianico, a red grape variety cultivated predominantly in Southern Italy and characterized by a particularly rich polyphenolic composition. The GPE used corresponds to the digestion extract obtained from Aglianico grape pomace of Cantine del Notaio (Rionero in Vulture, Potenza, Italy), previously characterized by LC-HRMS and LC-HRMS/MS-based molecular networking [11], which identified catechin, epicatechin, quercetin, petunidin-3-O-glucoside, malvidin-3-O-glucoside, delphinidin-3-O-glucoside, and procyanidin B2 as major specialized metabolites, with no detectable stilbenes. Aglianico grape pomace contains bioactive molecules with recognized antioxidant and chemopreventive properties, and eco-friendly extraction techniques have been developed to optimize recovery of its specialized metabolites [11].

Grape pomace-derived polyphenols have been shown to exert cardioprotective effects in experimental models of myocardial ischemia, with antioxidant and anti-inflammatory activities supporting their potential as adjuvant therapies in cardiovascular pathologies characterized by oxidative stress [12,13]. However, the direct electrophysiological effects of GPE on cardiac pacemaker activity have not been previously explored.

To address this gap, we used a multispecies approach combining HL-1 cardiomyocytes and *Drosophila melanogaster*. Electrophysiological experiments were performed in HL-1 cardiomyocytes, an immortalized atrial cardiomyocyte cell line derived from the AT-1 mouse atrial tumor lineage that retains differentiated cardiac morphological, biochemical, and electrophysiological properties and has been validated for studies of cardiac ion channel pharmacology [14]. Importantly, HL-1 cells express I_f_ and exhibit spontaneous pacemaker-like activity, making them a suitable cellular model for investigating HCN channel modulation. To extend our findings to a more integrated biological preparation, we additionally used the *D. melanogaster* larval heart tube, a myogenic, autonomic-nervous-system-independent cardiac model with conserved molecular–genetic mechanisms of cardiac development, widely used for pharmacological studies of cardiac rhythm [15].

Our results demonstrate that chronic treatment with Aglianico GPE reduces spontaneous action potential firing rate in HL-1 cells, selectively prolongs the diastolic depolarization phase of action potentials, reduces I_f_ density without altering its voltage dependence, and slows spontaneous beating rate in the *Drosophila* heart tube. Rescue experiments with isoproterenol and intracellular cAMP dialysis suggest that GPE interferes with HCN channel function through a mechanism distinct from all previously characterized natural modulators of I_f_, with evidence pointing to a cAMP-independent component of action. These findings identify Aglianico GPE as a novel modulator of cardiac pacemaker activity and provide a mechanistic framework for understanding how grape-derived polyphenols may influence cardiac automaticity.

## 2. Materials and Methods

Grape pomace extract (GPE)

Grape pomace from the Lucanian cultivar Aglianico (*Vitis vinifera* L.) was collected in 2019 from “Cantine del Notaio” winery (Rionero in Vulture, Potenza, Italy) after fermentation (22–26 °C, 6 days, RT) and the subsequent racking step. The pomace was dried in an oven at 55 °C for 6 h, reaching a final moisture content of 2.05 ± 0.91%. For extraction, 15 g of dried pomace was mixed with a hydroalcoholic solution (50% ethanol/water) at a 1:10 raw material–solvent ratio and processed at 60 °C under continuous stirring for 2 h. The resulting extract (GPE) was handled as previously described by Ponticelli et al. [11], yielding an extraction efficiency of 11.45 ± 1.01%. The GPE was stored in the dark until further use.

Cell culture

The HL-1 cardiac muscle cell line (SCC065, Sigma-Aldrich, Saint Louis, MO, USA) was maintained in Claycomb Medium (51800C, Sigma-Aldrich), supplemented with 10% fetal bovine serum (F2442, Sigma-Aldrich), 1% penicillin–streptomycin (100 U/mL; 100 μg/mL) (P4333, Sigma-Aldrich), 2 mM L-glutamine (G7513, Sigma-Aldrich), and 0.1 mM norepinephrine [(±)-arterenol] (A0937, Sigma-Aldrich) in a 37 °C incubator with 5% CO_2_. Cells were cultured and seeded onto supports pre-coated with gelatin-fibronectin (5 μg/mL fibronectin, F1141, Sigma-Aldrich, in 0.02% bovine skin gelatin, G9391, Sigma-Aldrich) for 1 h in a 37 °C incubator with 5% CO_2_. Cell concentration and viability were assessed using 0.4% trypan blue staining and the LUNA-II™ automated cell counter (Logos Biosystems, Gunpo, Republic of Korea).

Cell viability assay

The potential cytotoxicity of GPE on HL-1 cardiomyocytes was evaluated using PrestoBlue^TM^ cell viability assay (Invitrogen^TM^ Carlsbad, CA, USA), in accordance with the manufacturer’s protocol. Cells were seeded in 96-well plates at a density of 1.0 × 10^4^ cells/well. After complete attachment, cells were treated with different concentrations (10 µg/mL, 25 µg/mL, 100 µg/mL, and 1 mg/mL) of GPE in complete growth medium for 48 h in a humidified incubator (37 °C, 5% CO_2_). Cells in complete medium without treatment served as the healthy control, and the medium was refreshed every 24 h during incubation. Additionally, cells were treated overnight with 2.5 µM staurosporine as a positive control for cell death prior to the viability assay. At the end of the 48 h treatment with GPE, PrestoBlue^TM^ reagent (1:10 dilution in complete medium) was added to the cells, and the plate was incubated for 2 h at 37 °C in the dark. Fluorescence (Ex: 544 nm; Em: 590 nm) was measured using a Fluostar^®^ Omega microplate reader (BMG LABTECH, Ortenberg, Germany). The cytotoxicity of GPE was determined by statistically comparing the fluorescence values of treated cells with those of untreated control cells.

Cytosolic Ca^2+^ measurements

Cytosolic Ca^2+^ measurements in HL-1 cardiomyocytes were performed as previously described [16,17]. Briefly, cells were seeded onto gelatin/fibronectin-coated 15 mm Ø glass coverslips. After complete cell adhesion, the cells were treated with 25 μg/mL GPE in complete Claycomb medium for 48 h or maintained in complete medium without treatment as controls. During the treatment, the cells were kept in a humidified incubator (5% CO_2_) at 37 °C. At the end of the treatment, cardiomyocytes were loaded with 6 μM FURA-2AM (Invitrogen, Carlsbad, CA, USA) in medium at 37 °C for 30 min immediately before the experiment. After dye loading, the cells were washed with extracellular solution and then maintained in the same solution composed of 138 mM NaCl, 4 mM KCl, 1 mM MgCl_2_, 10 mM HEPES, 10 mM glucose, and 1.8 mM CaCl_2_, pH 7.4, for the duration of the experiment. Coverslips with dye-loaded cells were mounted in a perfusion chamber (RC quick release non-magnetic chamber, Warner Instruments, Hamden, CT, USA) housed in a Quick Exchange Platform (QE-1, Warner Instruments). Measurements were performed using an inverted microscope (Nikon Eclipse TE2000-S, Nikon, Tokyo, Japan) equipped for single-cell fluorescence measurements and imaging analysis. The sample was illuminated through a 40× oil immersion objective (Numerical Aperture = 1.30). The FURA-2AM-loaded cells were excited at 340 nm and 380 nm every 5 s. Emitted fluorescence was passed through a dichroic mirror, filtered at 510 nm (Omega Optical, Brattleboro, VT, USA), and captured by a cooled CCD camera (CoolSNAP HQ, Photometrics, Tucson, AZ, USA). Fluorescence recordings were performed using Metafluor software (Version 7.7.3.0, Molecular Devices, San Jose, CA, USA). The results are presented as the ratio of fluorescence acquired (340/380 nm) and normalized to the basal fluorescence ratio observed in the absence of a stimulus (R/R_0_). The experiments were conducted at room temperature. To evaluate ER Ca^2+^ levels, cells were stimulated with 10 mM caffeine (Sigma-Aldrich) to induce RyR-mediated Ca^2+^ release in a calcium-free extracellular solution with the addition of 50 µM EGTA (Sigma-Aldrich). To determine the intracellular Ca^2+^ concentration, Fura-2AM traces were corrected for background fluorescence, and the fluorescence ratio (R) was calibrated according to the following equation: [Ca^2+^]_i_ = Kd × Q(R − Rmin)/(Rmax − R), where Kd (224 nM) represents the dissociation constant of Fura-2AM for Ca^2+^_i_, and Q indicates the ratio of fluorescence intensities at minimum and maximum Ca^2+^ concentrations at 380 nm. Calibration of each sample was performed by adding 5 µM ionomycin in the presence of 0.5 mM EGTA (Rmin), followed by 5 µM ionomycin in 10 mM CaCl_2_ (Rmax). Data are expressed as means ± SEM, with n equal to the number of cells.

Electrophysiological Recordings

Electrophysiological recordings on HL-1 cardiomyocytes were performed as previously reported [18]. Patch-clamp experiments in whole-cell configuration were conducted with a Multiclamp 700B amplifier (Axon CNS-Molecular Devices, Sunnyvale, CA, USA), interfaced with the Axon Digidata 1500 (Axon Instruments-Molecular Devices, Sunnyvale, CA, USA). Data acquisition and analysis were carried out using Axon AXoScope 10.4 (Molecular Devices, Sunnyvale, CA, USA) and Axon pClamp 10.4 (Molecular Devices) software, with data sampled at 10 kHz and low-pass filtered at 5 kHz. For the patch-clamp experiments, HL-1 cardiomyocytes were seeded onto gelatin/fibronectin-coated 35 mm Ø Petri dishes and maintained in culture in a humidified incubator (5% CO_2_) at 37 °C until the analysis. After complete adhesion, the cardiomyocytes were treated with 25 μg/mL GPE in complete Claycomb medium for 48 h or maintained in complete medium without treatment as controls. The experiments were conducted at room temperature under optical control using an Olympus B51WI microscope (Olympus, Tokyo, Japan). Recording electrodes were pulled with a P-1000 puller (Sutter Instruments, Novato, CA, USA) from borosilicate glass pipettes (O.D.: 1.5 mm, I.D.: 0.86 mm, Sutter Instruments) to achieve resistances of 3–4 MΩ when filled with the pipette solution. Spontaneous action potentials (APs) were recorded in current–clamp (CC) mode, without current injection, using an intracellular solution containing (in mM): 144 KCl, 2 MgCl_2_, 10 HEPES, 5 EGTA (pH 7.2 and osmolarity 280 ± 10 mOsm/kg), and an extracellular solution containing (in mM): 138 NaCl, 4 KCl, 1 MgCl_2_, 1.8 CaCl_2_, 10 HEPES, 10 glucose (pH 7.4 and osmolarity 290 ± 10 mOsm/kg). APs were analyzed for the following parameters: frequency (Hz), cycle length (CL, ms), action potential duration at 100% repolarization (APD_100_), diastolic depolarization phase (DDP) duration (ms) and amplitude (mV), maximum diastolic potential (MDP, mV), threshold (mV), and amplitude (mV). The same intracellular solution was used to record membrane currents elicited by depolarizing voltage steps ranging from −80 mV to +30 mV, from a holding potential of −100 mV. Peak inward and outward currents for each cell were normalized to cell capacitance and plotted against membrane potential (V_m_) to obtain current–voltage (I–V) relationships. The I_f_, mediated by HCN channels, was isolated using an intracellular solution containing (in mM): 120 K+-Asp, 10 TEA-Cl, 2 MgCl_2_, 11 EGTA, 5 CaCl_2_, 10 HEPES (pH 7.2), and an extracellular solution containing (in mM): 140 NaCl, 30.4 KCl, 1.8 CaCl_2_, 1.2 MgCl_2_, 10 glucose, 5 HEPES, 2 NiCl_2_, 2 BaCl_2_, 0.5 4-AP (pH 7.35). When required, 100 µM isoproterenol was added to the extracellular solution in the recording chamber 10 min before starting the recording to enhance cAMP concentration in the cardiomyocytes via β-adrenergic stimulation. Additionally, in some recordings, 50 µM 8-Br-cAMP was directly added to the intracellular pipette solution. I_f_ was recorded in voltage-clamp (VC) mode using a hyperpolarization protocol consisting of 10 mV steps from −60 mV to −150 mV with a holding potential of −40 mV. Following the hyperpolarizing steps, an additional step to +40 mV was applied to facilitate channel closing. The I_f_ current measured at the end of each hyperpolarizing step was normalized to the cell capacitance (pA/pF) and plotted against the V_m_ to obtain the I/V plot. The normalized current evoked at each hyperpolarizing step was divided by the maximal current to construct the activation curve. Activation curves were fitted to a Boltzmann equation to obtain the slope factor (K) and the half-maximal activation voltage (V½) of the current.

cAMP Measurement

Intracellular cAMP levels in HL-1 cells were quantified using the cAMP Complete ELISA Kit (Enzo Life Sciences, Farmingdale, NY, USA; ADI-900-163A) following the non-acetylated protocol. Cells were seeded in 12-well culture plates at a density of 1 × 10^5^ cells per well. After complete adhesion, the cells were treated with 25 µg/mL GPE for 48 h or maintained in culture medium for the same period as a control. Two control conditions were established: (1) untreated cells to assess basal cAMP levels in the cytosol, and (2) cells stimulated for 10 min immediately before analysis with 100 µM IBMX and 5 µM FSK, serving as a technical control to ensure maximal cAMP accumulation and verify proper assay performance. At the end of the treatments, cells were lysed by incubating for 10 min in lysis buffer (0.1 M HCl, Enzo) supplemented with 0.5% Triton X-100 while continuously shaking. After lysis, cell samples were centrifuged at 600× *g* to remove cellular debris, and the supernatant was collected and immediately analyzed according to the manufacturer’s instructions. At the end of the assay, the signal from each sample was measured using a spectrophotometer at 405 nm. cAMP concentrations were calculated using a 4-parameter logistic (4PL) curve fitting program (Prism version 8, GraphPad Software, San Diego, CA, USA) and cAMP values were normalized to total protein concentrations determined by the Bradford assay (Bio-Rad, Hercules, CA, USA).

*Drosophila* husbandry

*Drosophila melanogaster w*^1118^ were maintained at 25 °C on standard cornmeal–agar medium, under a 12 h light/12 h dark cycle. Stocks were regularly transferred to fresh food vials to prevent overcrowding. For dietary exposure, freshly eclosed adults were transferred to vials containing food supplemented with 100 µg/mL GPE or control medium. Mixed sex parental groups were allowed to mate and lay eggs on the respective media for 24 h. After this period, adults were removed, and the developing progeny were maintained on the same food until the wandering third instar larval stage.

Monitoring larval *Drosophila* heartbeat

Third instar larvae were collected for optical recording of heart contractions as described previously [19]. In brief, the larvae were positioned dorsal side up using cyanoacrylate glue. Heart contractions were recorded for five minutes using a camera mounted on the microscope, and the heart rate was determined by manually counting the number of beats per minute (BPM) in slowed-down videos to facilitate accurate observation of heartbeats.

Statistical analysis

Statistical analysis and graphing of the data were performed using GraphPad Prism 8. The data are presented as the mean ± standard error of the mean (SEM), derived from pooled results across multiple experimental days. Statistical significance was determined using one-way or two-way ANOVA, or Student’s *t*-test for unpaired data, as appropriate to the dataset. A *p*-value of <0.05 (*) was considered statistically significant. In the context of these experiments, n indicates the number of cells/larvae analyzed, while ‘N’ represents the number of experimental replicates.

## 3. Results

### 3.1. GPE Reduces Spontaneous Firing and Selectively Prolongs the Diastolic Depolarization Phase in HL-1 Cardiomyocytes

**Figure 1:**
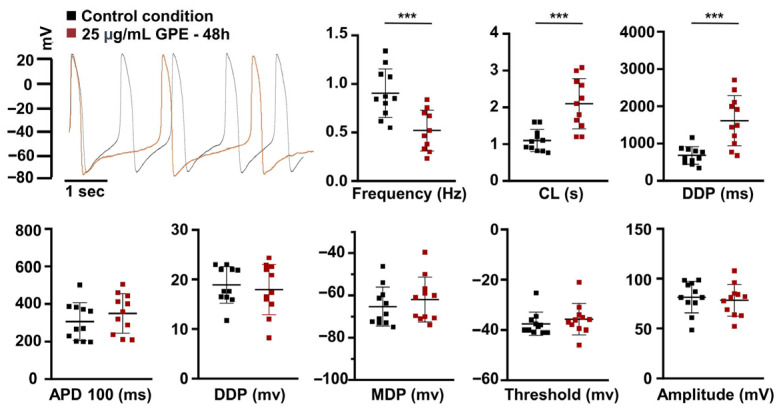
Analysis of the APs in HL-1 cardiomyocytes. Representative AP recordings by whole-cell patch clamp in the current–clamp configuration in HL-1 control cells (black trace) and HL-1 GPE cells (red trace) and scatter plots for the analysis of AP parameters in control condition (black dots, *n* = 11) and after GPE treatment (red squares, *n* = 11). After 48 h of treatment with 25 µg/mL GPE, the frequency of action potential (AP) firing decreased significantly (Hz: 0.51 ± 0.06) compared to control cardiomyocytes (Hz: 0.90 ± 0.07, *p*-value: 0.0006). In addition, the AP cycle length was longer in GPE-treated cells (2.09 ± 0.20 s, *n* = 11) compared to control cells (1.09 ± 0.09 s, *n* = 11; *t*-test *p*-value: 0.0003). The diastolic depolarization (DDP) duration in GPE-treated cells increased significantly by about 52% compared to control cells (680.7 ms ± 70.81, *n* = 11 vs. 1613 ms ± 202.9, *n* = 11; *t*-test *p*-value: 0.0003). No significant changes were observed in the APD100 (control cells: 306 ms ± 30.16, *n* = 11; GPE-treated cells: 349.5 ms ± 31.52, *n* = 11), DDP amplitude (control cells: 18.88 mV ± 1.11, *n* = 11; GPE-treated cells: 17.97 mV ± 1.53, *n* = 11), MDP (control cells: −65.31 mV ± 2.77, *n* = 11; GPE-treated cells: −62 mV ± 3.18, *n* = 11), threshold (control cells: −37.51 mV ± 1.38, *n* = 11; GPE-treated cells: −35.71 mV ± 1.87, *n* = 11), and amplitude (control cells: 81.46 mV ± 4.76, *n* = 11; GPE-treated cells: 78.29 mV ± 4.77). Data are presented as mean ± SEM. Student’s *t*-test for unpaired data ***: *p*-value ≤ 0.001.

To assess the effect of long-term treatment with GPE on membrane electrical properties, we recorded spontaneous action potentials (APs) from HL-1 cardiomyocytes treated with 25 µg/mL GPE for 48 h and compared them to untreated controls using whole-cell patch-clamp experiments. The selected concentration was preliminarily assessed by means of the PrestoBlue assay (Supplementary Material Figure S1). Notably, this concentration lies within the range commonly used in cardiomyocyte in vitro studies involving grape-derived polyphenols (1–25 µg/mL), where biological activity without cytotoxic effects has been consistently reported [20]. As shown in Figure 1, GPE treatment significantly reduced AP frequency (0.51 ± 0.06 Hz, *n* = 11) compared to controls (0.90 ± 0.07 Hz, *n* = 11, *p*-value = 0.0006, *t*-test). To characterize the impact of GPE on the various phases of the AP, we analyzed the following parameters: cycle length (CL, defined as the time between successive thresholds), action potential duration at 100% repolarization (APD100, measured from threshold to maximum diastolic potential), the diastolic depolarization phase (DDP, defined as the time from maximum diastolic potential [MDP] to the next threshold), the MDP, the threshold, and the amplitude (defined as the difference in mV between the AP peak and the MDP). The reduction in frequency was associated with a significant increase in both CL (control: 1.09 ± 0.09 s, *n* = 11; GPE: 2.09 ± 0.20 s, *n* = 11; *t*-test *p* = 0.0003) and DDP duration (control: 680.7 ± 70.81 ms, *n* = 11; GPE: 1613 ± 202.9 ms, *n* = 11; *t*-test *p* = 0.0003). No statistically significant differences were detected in APD100 (control: 306 ± 30.16 ms, *n* = 11; GPE: 349.5 ± 31.52 ms, *n* = 11), DDP amplitude (control: 18.88 ± 1.11 mV, *n* = 11; GPE: 17.97 ± 1.53 mV, *n* = 11), MDP (control: −65.31 ± 2.77 mV, *n* = 11; GPE: −62.00 ± 3.18 mV, *n* = 11), threshold (control: −37.51 ± 1.38 mV, *n* = 11; GPE: −35.71 ± 1.87 mV, *n* = 11), or AP amplitude (control: 81.46 ± 4.76 mV, *n* = 11; GPE: 78.29 ± 4.77 mV). The selective prolongation of the DDP is consistent with a modulation of currents contributing to this phase, including HCN-dependent inward current and, potentially, NCX-mediated depolarizing current [3,21].

### 3.2. GPE Does Not Alter Depolarization-Activated Voltage-Dependent Currents in HL-1 Cardiomyocytes

**Figure 2:**
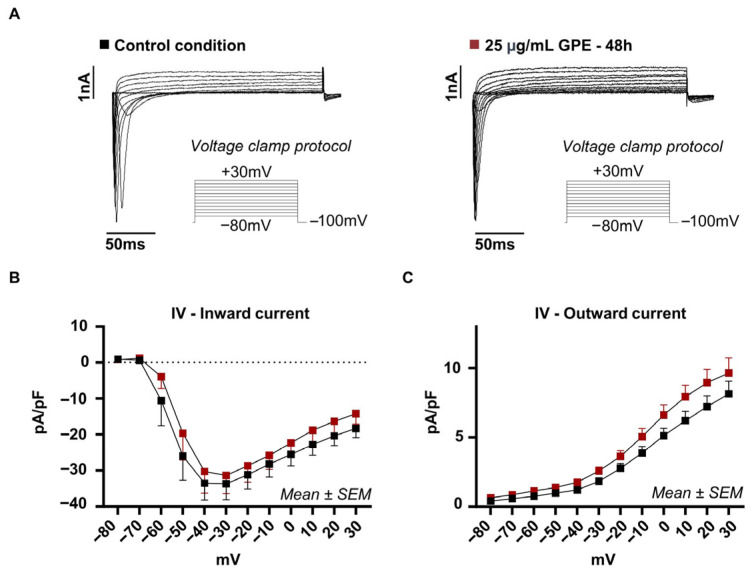
Study of inward and outward currents in HL-1 cardiomyocytes. (**A**) Representative traces of inward and outward currents recorded in HL-1 cells treated with 25 µg/mL GPE for 48 h (right) and in control cardiomyocytes (left) are shown in the figure. The recorded ionic currents were evoked using a voltage clamp protocol consisting of a holding potential of −100 mV and depolarization steps in 10 mV increments from −80 mV to 30 mV. (**B**,**C**) Analysis of the recorded currents, normalized to membrane capacitance (pA/pF) and presented as current–voltage (I–V) relationships, shows the presence of inward (**B**) and outward (**C**) currents of comparable amplitude and kinetics under both experimental conditions. Specifically, in cells treated with GPE 25 µg/mL for 48 h, a peak inward current at −30 mV of −33.6 ± 4.4 pA/pF (*n* = 11) and a maximum outward current at 30 mV of 8.1 ± 3 pA/pF (*n* = 11) were recorded. Similarly, control HL-1 cells showed a peak inward current at −30 mV of −31.3 ± 5.0 pA/pF and a maximum outward current at 30 mV of 9.6 ± 3.7 pA/pF (*n* = 11).

To further substantiate the absence of changes in APD100 and AP amplitude observed under current–clamp conditions, we examined depolarization-activated inward and outward currents under voltage clamp (Figure 2A). GPE-treated cells (25 µg/mL, 48 h; *n* = 11) showed a peak inward current of −33.6 ± 4.4 pA/pF at −30 mV (Figure 2B) and a maximum outward current of 8.1 ± 3.0 pA/pF at +30 mV (Figure 2C), comparable to control cells (peak inward: −31.3 ± 5.0 pA/pF; maximum outward current of 9.6 ± 3.7 pA/pF; *n* = 11), with no statistically significant differences between groups. These findings indicate that chronic GPE exposure does not significantly modify depolarization-activated inward or outward conductances, supporting a selective effect on currents governing diastolic depolarization rather than a generalized alteration of membrane excitability.

### 3.3. GPE Does Not Affect Cytosolic Ca^2+^ Level in HL-1 Cardiomyocytes

**Figure 3:**
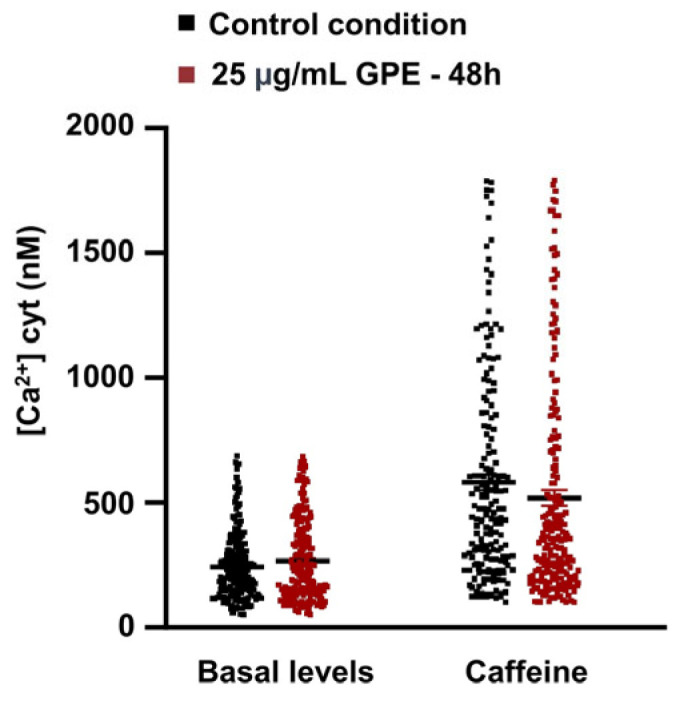
Effects of 25 µg/mL GPE on [Ca^2+^] cyt in HL-1 cardiomyocytes in basal condition or during caffeine stimulation. [Ca^2+^] cyt was determined using the cytosolic calcium-sensitive fluorescent probe Fura-2 AM. The scatter plot shows that after 48 h of treatment, GPE (red square, [Ca^2+^] cyt: 260 nM ± 10.39, *n* = 236) does not affect basal calcium levels compared to the control condition (black square, [Ca^2+^] cyt: 242 nM ± 9.27, *n* = 214; ctr vs. GPE *t*-test: *p*-value 0.22). Similarly, the cytosolic calcium concentration measured during stimulation with caffeine (10 mM) does not change between the two analyzed conditions (control: black square, [Ca^2+^] cyt: 581 nM ± 31.65, *n* = 178; GPE: red square, [Ca^2+^] cyt: 518.2 nM ± 30.31, *n* = 212; ctr vs. GPE, *t*-test: *p*-value 0.15). The data collected suggest that 25 µg/mL of GPE does not affect cytosolic calcium dynamics after 48 h of treatment. Data are presented as mean ± SEM.

In the late phase of diastolic depolarization, the Na^+^/Ca^2+^ exchanger (NCX) facilitates the efflux of intracellular Ca^2+^ in exchange for the influx of extracellular Na^+^, driven by the electrochemical gradient for Na^+^ across the plasma membrane. Operating at a stoichiometry of 3 Na^+^ ions per Ca^2+^ ion, this electrogenic process generates an inward depolarizing current that contributes to cardiac automaticity in sinoatrial node pacemaker cells [21,22]. NCX activity is tightly coordinated with the sarcoplasmic reticulum, as Ca^2+^, itself the primary activating factor for NCX, is largely provided by sarcoplasmic reticulum Ca^2+^ release, which drives NCX current activation during late diastole [23,24]. To assess whether GPE affects NCX activity, we measured cytosolic Ca^2+^ levels using the fluorescent probe Fura-2 AM as an indirect indicator of NCX functionality in HL-1 cardiomyocytes treated with 25 µg/mL GPE for 48 h, compared to untreated controls. Measurements were taken both at rest and during caffeine-induced sarcoplasmic reticulum Ca^2+^ release. As shown in Figure 3, GPE-treated HL-1 cells showed basal Ca^2+^ levels comparable to controls (controls: 242.8 ± 9.27 nM, *n* = 214; GPE: 260 ± 10.39 nM, *n* = 236), and no significant differences were observed during caffeine-induced sarcoplasmic reticulum Ca^2+^ release (controls: 581 ± 31.65 nM, *n* = 178; GPE: 518.2 ± 30.31 nM, *n* = 212). The absence of detectable changes in cytosolic Ca^2+^ homeostasis argues against major alterations in global Ca^2+^ handling and makes a predominant contribution of NCX to the observed diastolic slowing less likely.

### 3.4. GPE Reduces I_f_ in HL-1 Cardiomyocytes

**Figure 4:**
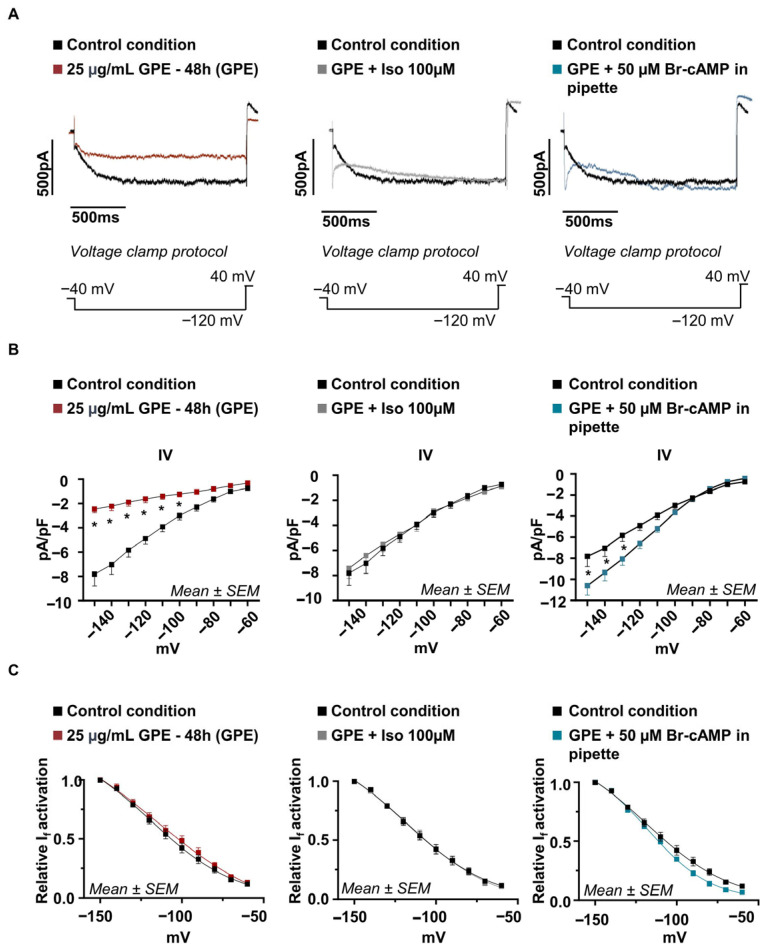
Analysis of the Funny current (I_f_) in HL-1 cardiomyocytes. (**A**) Representative traces of the isolated I_f_ current at −120 mV under each experimental condition: on the left, a comparison between cells treated for 48 h with GPE 25 µg/mL (in red) and the control condition (in black); in the center, a comparison between cells treated for 48 h with GPE 25 µg/mL in the presence of 100 µM isoproterenol and the control condition; on the right, a comparison between cells treated for 48 h with GPE 25 µg/mL in the presence of 50 µM 8-Br-cAMP in the patch pipette and the control condition. (**B**) Analysis of the I_f_ current recorded with hyperpolarizing current steps from −60 mV to −150 mV (with 10 mV increments, holding at −40 mV), normalized to membrane capacitance (pA/pF) and presented in a current–voltage (I–V) plot. Two-way ANOVA analysis showed (left) a significant reduction in the current recorded in cells treated with GPE 25 µg/mL for 48 h from −150 mV to −100 (red square, *n* = 9; at −120 mV: −1.62 ± 0.30 pA/pF) compared to the control condition (black square, *n* = 8; at −120 mV: −4.89 ± 0.45 pA/pF; *p*-value at −120 mV < 0.05). A significant recovery of the current amplitude is observed in cells treated with GPE 25 µg/mL for 48 h following the addition of 100 µM isoproterenol in the extracellular solution (gray square, *n* = 8; at −120 mV: −4.7 ± 0.51 pA/pF; graph in the center) or 50 µM 8-Br-cAMP in the patch pipette (blue square, *n* = 5; at −120 mV: −6.5 ± 0.47 pA/pF; graph on the right). Data presented as mean ± SEM; two-way ANOVA *: *p*-value ≤ 0.05. (**C**) The activation curves (relative I_f_ activation) obtained by fitting the I/Imax ratio of the current to a Boltzmann function. The analysis of the activation curves reveals comparable activation kinetics in control and treated cells, both in the absence and presence of isoproterenol, or following the addition of 8-Br-cAMP in the patch pipette. Specifically, control cells exhibited a V½ of −115 ± 2.7 mV and a slope (K) of 24.07 ± 2.24. Similarly, cells treated with 25 µg/mL GPE for 48 h (left) showed a V½ of −108.9 ± 4.6 mV and an activation curve slope (K) of 22.22 ± 2.57 mV; while GPE-treated cells analyzed in the presence of isoproterenol in the extracellular solution (center) exhibited a V½ of −109.8 ± 2.5 mV and a K of 24.14 ± 2.00 mV. GPE-treated cells analyzed in the presence of cAMP in the patch pipette (right) showed a V½ of −117.4 ± 1.07 mV and a K of 19.8 ± 1.6 mV. Data presented as mean ± SEM; no significant differences were found in the *t*-test for V½ and K values when comparing the three experimental conditions to the control condition.

In the early phase of diastolic depolarization, hyperpolarization activates HCN channels, allowing the simultaneous passage of Na^+^ and K^+^ ions and generating the inward depolarizing I_f_, a key determinant of spontaneous cardiac activity and rate control [3]. To assess the impact of GPE on I_f_, the current was pharmacologically isolated in HL-1 cardiomyocytes treated with 25 µg/mL GPE for 48 h and compared with untreated controls using whole-cell patch-clamp recordings. As shown in Figure 4, GPE treatment significantly reduced I_f_ current density at strongly hyperpolarizing potentials (−100 to −150 mV). At −120 mV (Figure 4A), current density was −1.62 ± 0.30 pA/pF in GPE-treated cells (*n* = 9) compared with −4.89 ± 0.45 pA/pF in controls (*n* = 8; *p* < 0.05). Although such voltages exceed the physiological diastolic range, they are routinely used to assess near-maximal HCN channel activation and reliably quantify current density; the complete I–V relationship and normalized activation curves (I/I_max_) are shown in Figure 4, in the B and C panels, respectively. To determine whether this reduction involved altered cAMP-dependent gating, we increased intracellular cAMP concentration by two independent means. We either activated endogenous β-adrenergic receptors by applying 100 µM isoproterenol or dialyzed a cell-permeable cAMP, including (50 µM 8-Br-cAMP) directly in the patch pipette; both maneuvers were applied to GPE-treated cells. In HCN channels, increases in intracellular cAMP enhance channel open probability and shift voltage dependence toward more positive potentials [2]. In GPE-treated cells, both isoproterenol (−4.7 ± 0.51 pA/pF at −120 mV; *n* = 8) and 8-Br-cAMP (−6.5 ± 0.47 pA/pF at −120 mV; *n* = 5) significantly increased I_f_ amplitude compared with basal GPE conditions, indicating that cAMP-dependent modulation is preserved (Figure 4A). Consistently, normalized activation curves revealed no statistically significant differences in V½ or slope factor (K) across conditions (control: V½ = −115 ± 2.7 mV, K = 24.07 ± 2.24; GPE: V½ = −108.9 ± 4.6 mV, K = 22.2 ± 2.5; GPE + isoproterenol: V½ = −109.8 ± 2.5 mV, K = 24.14 ± 2; GPE + 8-Br-cAMP: V½ = −117.4 ± 1.07 mV, K = 19.8 ± 1.6) (Figure 4B,C). Consistently, in untreated HL-1 cells, isoproterenol alone significantly increased I_f_ amplitude from −150 mV to −100 mV (at −120 mV: from −4.89 ± 0.45 to −10.10 ± 1.11 pA/pF; *n* = 6) without producing a significant shift in V½ (Supplementary Figure S2). Although modest shifts cannot be entirely excluded, these data suggest that GPE reduces I_f_ density without markedly impairing cAMP-dependent channel modulation. To further clarify whether GPE directly affects HCN channel function or instead reduces global cAMP availability, cytosolic cAMP was subsequently quantified as described below.

### 3.5. GPE Does Not Affect cAMP Levels in HL-1 Cardiomyocytes

**Figure 5:**
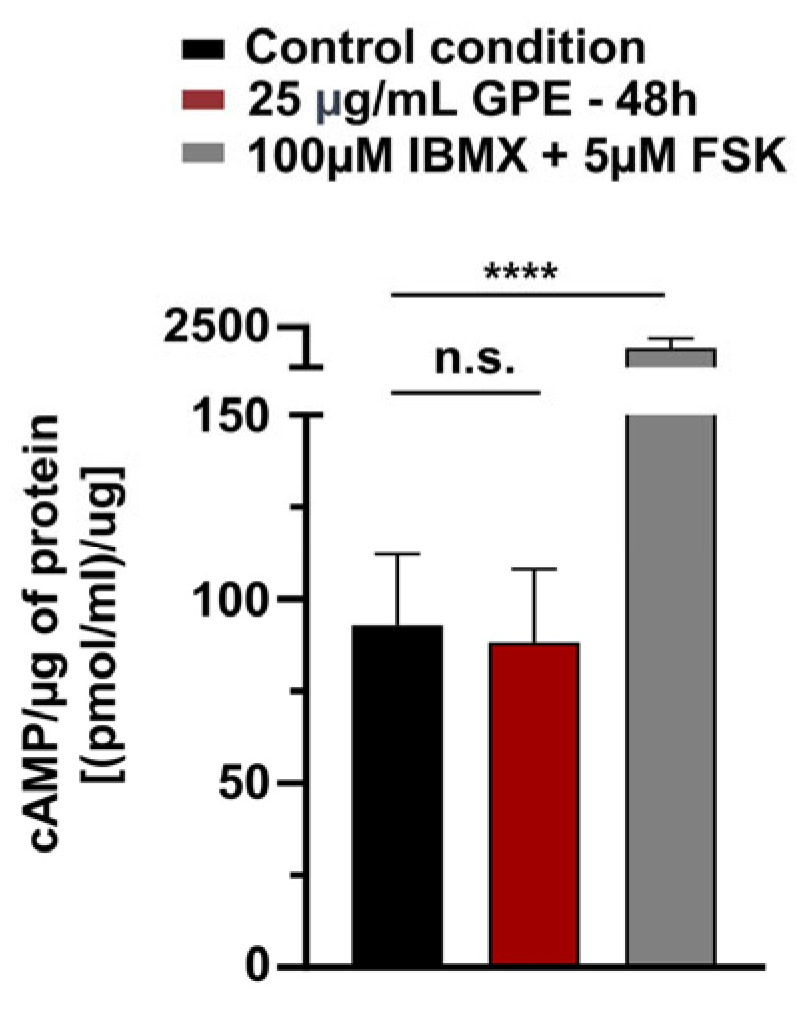
Quantification of cytosolic cAMP in HL-1 cardiomyocytes. ELISA experiments showed no significant differences in cAMP levels between the total cell lysates in GPE 48 h treated cells (N = 3, 88.33 ± 2.546 cAMP/µg protein) compared to control cells (N = 3, 92.94 ± 7.8 cAMP/µg protein; *t*-test *p*-value: 0.69). The treatment with IBMX 100 µM + FSK 5 µM for 10 min was used as internal control. Data are presented as mean ± SEM. Student’s *t*-test for unpaired data ****: *p*-value ≤ 0.0001; n.s.: not significant.

To further clarify whether the effect of GPE was mediated by a direct modulation of the HCN channel or indirectly by decreasing intracellular cAMP concentration we measured cytosolic cAMP in cells treated with GPE. Quantification of cytosolic cAMP by ELISA (Figure 5) revealed no significant difference between GPE-treated and control HL-1 cells (GPE-treated cells, N = 3, 88.33 ± 2.546 cAMP/µg protein; control cells, N = 3, 92.94 ± 7.8 cAMP/µg protein; *t*-test, *p* = 0.69). These data indicate that chronic GPE exposure does not produce detectable changes in basal global intracellular cAMP levels.

Taken together, these data suggest that the GPE-induced reduction in I_f_ current density is largely independent of cAMP signaling and mediated by other mechanisms affecting HCN channel function.

### 3.6. Acute Application of GPE Rapidly Reduces I_f_

**Figure 6:**
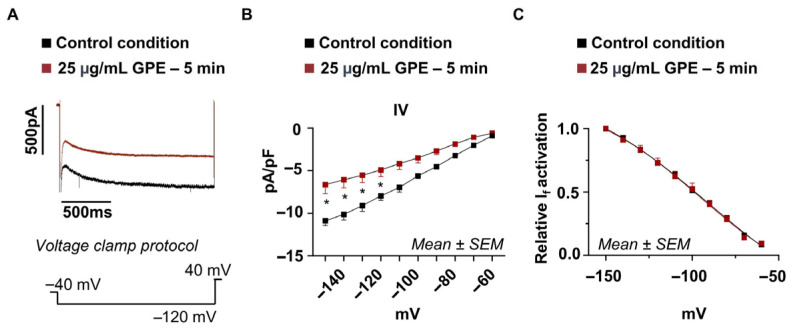
Acute effect of GPE on I_f_ in HL-1 cardiomyocytes. (**A**) Representative I_f_ traces recorded at −120 mV from a holding potential of −40 mV in the same HL-1 cell before (black) and after (red) acute bath application of 25 μg/mL GPE (~5 min after complete solution exchange). (**B**) Current–voltage (I–V) relationship of I_f_, normalized to membrane capacitance (pA/pF), in control conditions (black squares) and after acute GPE application (red squares). Two-way ANOVA analysis showed a significant reduction in the current recorded in cells treated with GPE 25 µg/mL under acute exposure from −150 mV to −110 mV (red square, *n* = 5; at −120 mV: −4.9 ± 0.78 pA/pF) compared to the control condition (black square, *n* = 5; at −120 mV: −7.9 ± 0.53 pA/pF; *p*-value at −120 mV < 0.001). Data are presented as mean ± SEM; two-way ANOVA *: *p*-value ≤ 0.05. (**C**) Normalized activation curves (I/Imax) obtained by fitting current activation to a Boltzmann function. No significant differences in V½ or slope factor (K) were detected between conditions. Data are presented as mean ± SEM.

To distinguish whether the GPE-induced reduction in I_f_ reflects a rapid action on HCN channels or requires longer-term cellular adaptations, we examined the temporal profile of GPE action by acute bath application. In each HL-1 cardiomyocyte, I_f_ was first recorded in control bath solution and then again after complete bath exchange with solution containing 25 μg/mL GPE (~5 min after exchange onset). As shown in Figure 6, acute GPE exposure significantly reduced I_f_ amplitude at hyperpolarizing potentials. At −120 mV, current density was −7.9 ± 0.53 pA/pF under control conditions and −4.9 ± 0.78 pA/pF after acute GPE application, corresponding to a ~38% reduction in current amplitude (*n* = 5 cells). Normalized activation curves were superimposable in control and GPE conditions, with no detectable shift in the V½ or in the slope factor (K), indicating that acute GPE reduces I_f_ amplitude without altering the voltage dependence of channel gating, a profile consistent with that observed after 48 h of chronic exposure (Figure 4).

### 3.7. GPE Reduces Drosophila Heart Rate

**Figure 7:**
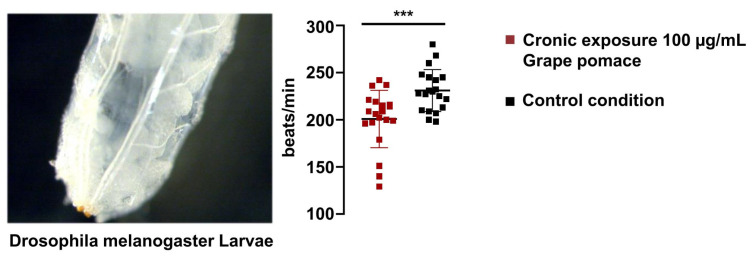
Effect of GPE (100 µg/mL) on the heart beating frequency of *Drosophila melanogaster* larvae. Representative image of a larva showing the cardiac tube (left panel). Right panel, following the administration of a diet containing 100 µg/mL of GPE to *Drosophila* larvae for 96 h, a significant reduction in the number of cardiac beats per minute was observed (200.9 ± 29.67, *n* = 20) in comparison to the control larvae (231.0 ± 21.72, *n* = 20, Student’s *t*-test for unpaired data: *** *p* < 0.005).

Next, to extend our analysis beyond in vitro mammalian cardiomyocytes and to explore whether the effect of GPE persists in a cardiac system that relies on different pacemaking mechanisms, we turned to an in vivo *Drosophila* melanogaster model. Larval heart rhythmicity in *Drosophila* is driven primarily by Ca^2+^ and K^+^ channels and is largely independent of cAMP modulation [25,26], providing a distinct physiological context in which to assess GPE’s action. This system therefore offers a strategic advantage: if GPE were to reduce heart rate here as well, such an effect would point to mechanisms not restricted to the HCN/cAMP axis. Flies laid eggs on GPE-supplemented medium containing 100 µg/mL GPE, and the progeny remained on this medium for 96 h from oviposition to the third instar larval stage. Following continuous GPE exposure, larvae displayed a significant reduction in heart rate (200.9 ± 29.67 bpm, *n* = 20) compared with controls (231.0 ± 21.72 bpm, *n* = 20; *p* < 0.005) (Figure 7). These findings indicate that dietary GPE can exert negative chronotropic effects even in a cardiac system that operates independently of cAMP, suggesting a broader physiological action.

## 4. Discussion

Plant-derived extracts are complex mixtures of bioactive compounds whose overall biological activity often results from synergistic, additive, or antagonistic interactions among multiple constituents, and cannot be predicted solely on the basis of the known properties of individual molecules [27]. Such synergistic interactions may lower the effective therapeutic dose and confer a multitargeted mechanism of action [28]. In the present study, we therefore evaluated the chronotropic effects of the Aglianico GPE as an integral extract, without attempting to isolate individual active components.

The Aglianico GPE used here was chemically characterized in our previous work [11], which identified seven major specialized metabolites: the flavan-3-ols catechin, epicatechin and procyanidin B2, the flavonol quercetin, and the anthocyanins petunidin-, malvidin- and delphinidin-3-O-glucoside; stilbenes, including resveratrol, were not detected. Among these constituents, quercetin is the only one for which direct activity on HCN channels has been documented [29], and is therefore the most plausible single contributor to the observed reduction in If: in heterologous systems, quercetin inhibits HCN current amplitude (IC50 ≈ 27 μM on HCN2, with preferential potency over HCN1 and HCN4), shifts the activation curve toward more hyperpolarized potentials, and slows deactivation kinetics. The structurally related flavonoid fisetin binds directly to the HCN2 cyclic nucleotide-binding domain [30], providing a structural rationale for an HCN-targeted action of this chemical class. No direct HCN-modulating activity has been reported, to our knowledge, for catechins, procyanidin B2, or anthocyanins, suggesting that quercetin is the dominant HCN-targeting component within the GPE, with additive or synergistic contributions of the other constituents likely.

The main finding of the present study is that Aglianico GPE exerts a negative chronotropic effect in vitro and in vivo and in two phylogenetically distant cardiac models: HL-1 atrial cardiomyocytes and the *Drosophila melanogaster* heart tube. In HL-1 cells, GPE reduced the frequency of spontaneous action potentials. Voltage-clamp analysis revealed that this effect is associated with a significant reduction in I_f_ current density, without affecting the voltage dependence of channel activation. This pattern of action shares a key feature with the selective I_f_ current blocker ivabradine, specifically the slowing of cardiac automaticity via I_f_ current reduction. However, it differs mechanistically, since ivabradine acts as an open-channel blocker, reducing current amplitude without altering voltage dependence through a use-dependent mechanism, while GPE reduces current density without affecting the half-maximal activation voltage (V½) [31,32]. Furthermore, the cytosolic cAMP content was not altered by GPE treatment, yet both extracellular application of isoproterenol and intracellular dialysis with 8-Br-cAMP significantly increased I_f_ amplitude, indicating that cAMP-dependent modulation is preserved. These findings suggest that GPE does not modulate HCN channel activity by altering the cAMP signaling cascade, but may instead interfere with the functional coupling between cAMP and the HCN channel through a mechanism independent of cAMP availability or responsiveness, possibly involving direct effects on channel gating.

Among the natural compounds reported to modulate I_f_, GPE displays a mechanistic profile that is distinct from all previously characterized agents. Berberine and Acehytisine both reduce I_f_ but produce shifts in the voltage dependence of channel activation [7,8], indicating a direct interaction with the channel gating machinery. GBE irreversibly suppresses HCN2 and HCN4 currents [9], a feature suggestive of stable pore occlusion. TMYX, the most mechanistically characterized natural I_f_ modulator, acts as a competitive antagonist of cAMP at the CNBD, producing a negative shift in V½ and an increase in maximal conductance [10]. GPE shares with TMYX the absence of direct channel block but differs critically in that it does not shift V½ and does not interfere with cAMP-dependent modulation or intracellular cAMP levels. This aspect places Aglianico GPE in a mechanistic category not previously described among natural modulators of cardiac pacemaker activity and raises the question of which aspect of HCN channel regulation is specifically targeted. However, given that HL-1 cells display limited sensitivity in detecting cAMP-dependent shifts in voltage dependence [33], this interpretation should be considered with caution.

The cellular mechanism underlying the GPE-induced reduction in I_f_ density remains to be fully elucidated. Two hypotheses are compatible with the present data. First, GPE may interfere with the functional coupling between cAMP and the CNBD of HCN channels without evidence of direct competition for the cAMP binding site at the concentrations tested. Under basal conditions, this would reduce the number of functionally available channels, manifesting as a reduction in current density without altering the intrinsic voltage dependence of gating. The rescue of I_f_ by intracellular dialysis with 8-Br-cAMP is consistent with this interpretation, as saturation of the CNBD with a non-hydrolyzable cAMP analog would be expected to overcome a partial functional impairment of the domain. Alternatively, GPE may reduce the number of HCN4 channels available at the plasma membrane, either by impairing channel trafficking or by reducing channel expression. A reduction in surface channel density would equally account for the observed decrease in I_f_ without a shift in V½. The rescue by 8-Br-cAMP does not distinguish between these two mechanisms, since cAMP has been shown to modulate HCN channel trafficking through competitive interactions at the CNBD, a mechanism demonstrated for neuronal HCN1 but likely conserved across isoforms given the high structural homology of the CNBD [34]. Acute application experiments provide a constraint on these alternatives: a significant fraction of the I_f_ reduction develops within the time required for complete bath exchange (~5 min), a timescale incompatible with channel trafficking, internalization, or expression-level changes. This indicates that GPE acts at least in part through a direct and rapid effect on HCN channels and/or on their immediate membrane environment, rather than through a slower remodeling of channel availability. The magnitude of the acute reduction (~38% at −120 mV; Figure 6) was nonetheless smaller than that observed after 48 h of chronic exposure (~67% at −120 mV; Figure 4), and we cannot exclude that additional contributions associated with chronic stimulation may also be at play. It is worth noting that HL-1 cardiomyocytes display a reduced sensitivity of I_f_ to cAMP compared to native sinoatrial node cells [33]. In this low-cAMP environment, a CNBD-competitive mechanism similar to that of TMYX would not be expected to produce a detectable shift in V½, since the channels already operate largely in the cAMP-unbound configuration. This intrinsic feature of HL-1 cells is also confirmed by our own data: isoproterenol application in untreated cells significantly increased I_f_ amplitude without producing a significant shift in V½ (Supplementary Materials Figure S2), in line with the predominantly density-mediated cAMP responsiveness of I_f_ reported for this cell line. Therefore, the absence of a V½ shift in our experiments does not exclude a CNBD-competitive mechanism.

Beyond the modulation of I_f_, the possible contribution of Ca^2+^-handling mechanisms to the GPE-induced chronotropic effect was also considered. Plant-derived extracts and natural products commonly exert negative chronotropic effects in cardiomyocytes through reduction in cytosolic Ca^2+^, modulation of L-type Ca^2+^ channels, and alteration of intracellular Ca^2+^ transients [35]. Such effects could in principle influence spontaneous activity through secondary modulation of NCX or sarcoplasmic reticulum Ca^2+^ release. However, chronic GPE treatment did not alter basal cytosolic Ca^2+^ levels as assessed by Fura-2AM ratiometric imaging, and no major effects on voltage-dependent currents were apparent in our voltage-clamp recordings, making a primary role of L-type Ca^2+^ channels unlikely. This consideration is particularly relevant in light of recent evidence that β-adrenergic acceleration of heart rate is mediated in parallel by PKA-dependent disinhibition of Cav1.3 via phosphorylation of Rad and by cAMP-dependent modulation of HCN4, with each arm capable of sustaining the chronotropic response independently [36]. Within this updated framework, the absence of changes in depolarization-activated inward currents, particularly in the low-threshold range (−50 to −40 mV) where Cav1.3 provides its distinctive contribution due to activation at potentials negative to Cav1.2 [37], together with the unaltered action potential threshold (which would be displaced toward more depolarized potentials by a functionally relevant reduction in Cav1.3) and the preserved cytosolic Ca^2+^ homeostasis, argue against a primary action of GPE on the Cav1.3 arm. These arguments are indirect, as our protocols did not pharmacologically isolate I_Ca,L_ or discriminate Cav1.3 from Cav1.2, and Cav1.3 also generates a dihydropyridine-sensitive persistent Na^+^ inward current active throughout the diastolic depolarization range that would require dedicated protocols to be resolved [38]. A concomitant effect of GPE on Cav1.3, therefore, cannot be formally excluded and remains an interesting question for future studies. In the same context, a possible contribution of NCX similarly remains open, as dedicated protocols to isolate INCX were not employed in the present study.

The observation that GPE reduces the beating rate of the *Drosophila* heart provides additional mechanistic insight and supports a cAMP-independent component of action. The *Drosophila* cardiac pacemaker is myogenic and, unlike the mammalian sinoatrial node, does not rely on cAMP signaling for rate regulation. Pharmacological and genetic evidence indicate that the cAMP-PKA pathway does not contribute to heart rate control in the fly, with pacemaking instead depending critically on Ca^2+^ and K^+^ currents [25,26]. Notably, *Drosophila* encodes a single HCN channel gene (DmIh) [39], the ortholog of mammalian HCN channels, whose product is expressed in the contractile myocytes of both the larval and adult cardiac tube [40]. The ability of GPE to slow the *Drosophila* heartbeat in this cAMP-independent context is therefore consistent with a direct effect on HCN channel availability, for instance, through modulation of channel trafficking or expression, rather than exclusively through interference with cAMP-CNBD coupling. Such a mechanism would also be compatible with the HL-1 data: a reduction in channel density without voltage-dependence shift, and a rescue by supraphysiological 8-Br-cAMP concentrations that would shift the gating of residual channels sufficiently to compensate for their reduced number. Taken together, the results from both model systems point to a cAMP-independent, possibly post-translational or trafficking-related mechanism as a primary target of GPE in cardiac pacemaking.

These findings should, however, be interpreted in light of two important limitations. First, the electrophysiological characterization was performed in HL-1 cells, an immortalized atrial cardiomyocyte line that, while retaining key features of cardiac automaticity, differs from native sinoatrial node myocytes in action potential morphology, HCN isoform composition, and cAMP sensitivity [33,41]. While consistent effects were also observed in *Drosophila*, whether GPE exerts comparable effects on I_f_ current in primary mammalian pacemaker cells remains to be established. Second, GPE is a complex mixture of bioactive constituents, and the molecular identity of the active component(s) responsible for the observed chronotropic and electrophysiological effects has not been determined. Fractionation and activity-guided isolation will be necessary to identify the effector molecule(s) and define their direct molecular target. From a therapeutic perspective, the identification of a plant-derived extract capable of reducing cardiac automaticity through a mechanism distinct from those of existing pharmacological agents, including ivabradine and the natural compounds acting via V½ shift, broadens the landscape of potential strategies for the management of inappropriate sinus tachycardia and sinus node dysfunction. The cAMP-independence suggested by the *Drosophila* data is particularly noteworthy, as it raises the possibility of chronotropic modulation in conditions where the cAMP-HCN axis is already compromised or pharmacologically saturated.

## Data Availability

The original contributions presented in the study are included in the article/Supplementary Material; further inquiries can be directed to the corresponding authors.

## Supplementary Materials

The following supporting information can be downloaded at https://www.mdpi.com/article/10.3390/life16050786/s1: Figure S1: Effect of GPE on cell viability in HL-1 cardiomyocytes; Figure S2: Effect of isoproterenol on If in control HL-1 cardiomyocytes.

## Abbreviations

The following abbreviations are used in this manuscript:

AP	Action potential
APD100	Action potential duration at 100% repolarization
CL	Cycle length
CNBD	Cyclic Nucleotide-Binding Domain
DDP	Diastolic depolarization phase
GBE	Ginkgo biloba extract
GPE	Grape pomace extract
HCN	Hyperpolarization-activated cyclic nucleotide-gated
I_f_	Funny current
MDP	Maximum diastolic potential
NCX	Na^+^/Ca^2+^ exchanger
TMYX	Tongmai Yangxin
