# Supplementary material for: Aglianico Grape Pomace Extract Reduces Cardiac Pacemaker Activity by Decreasing Hyperpolarization-Activated Current Density Independently of cAMP Signaling
Source: Life (Basel). 2026 May 8;16(5):786. doi: 10.3390/life16050786 (PMC13208127; doi:10.3390/life16050786)
Supplement: Supplementary file 1 [file life-16-00786-s001.zip › Figure S1.pdf]

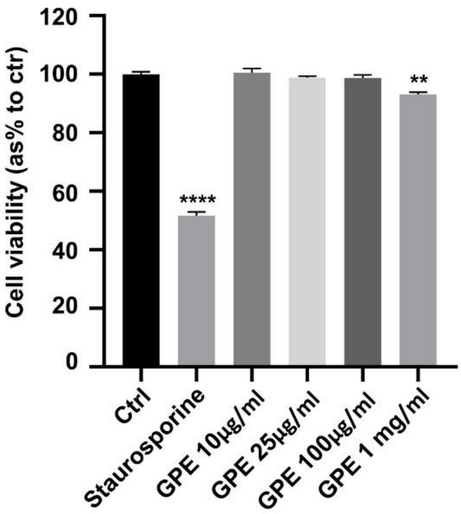

Figure S1. Effect of GPE on cell viability in HL-1 cardiomyocytes. Cell viability was assessed by PrestoBlue™ assay in HL-1 cardiomyocytes after 48 h of incubation with increasing concentrations of GPE (10 µg/mL to 1 mg/mL). Staurosporine (2.5 µM) was used as a positive control for cell death. Data are presented as mean ± SEM (N = 3 per condition). Statistical analysis was performed using one-way ANOVA compared to control cells. No significant differences were observed at 10, 25, or 100 µg/mL GPE compared to control, whereas 1 mg/mL GPE significantly reduced cell viability (**p = 0.0019). Staurosporine markedly decreased cell viability (****p < 0.0001).
