# Supplementary material for: Aglianico Grape Pomace Extract Reduces Cardiac Pacemaker Activity by Decreasing Hyperpolarization-Activated Current Density Independently of cAMP Signaling
Source: Life (Basel). 2026 May 8;16(5):786. doi: 10.3390/life16050786 (PMC13208127; doi:10.3390/life16050786)
Supplement: Supplementary file 1 [file life-16-00786-s001.zip › Figure S2_with figure legend.pdf]

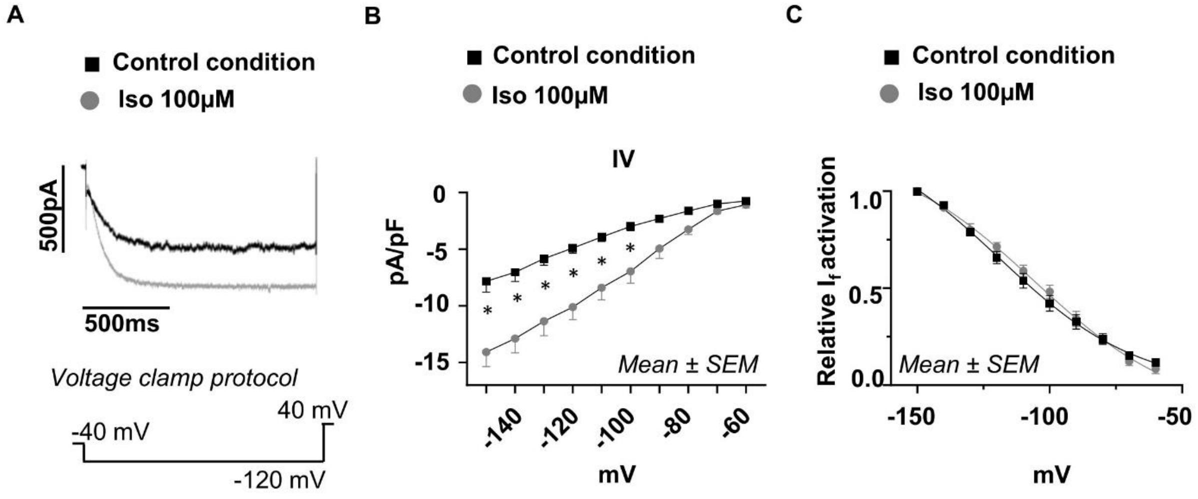

Figure S2. Effect of isoproterenol on I_f in control HL-1 cardiomyocytes. A. Representative I_f traces recorded at −120 mV from a holding potential of −40 mV in control HL-1 cells (in black) and after bath application of 100 µM isoproterenol (Iso, in grey). B. Current–voltage (I–V) relationship of I_f, normalized to membrane capacitance (pA/pF), in control conditions (black squares) and after Iso application (grey circles). Two-way ANOVA analysis showed a significant increase in I_f amplitude under Iso exposure from −150 mV to −100 mV (grey circles, n = 6; at −120 mV: −10.10 ± 1.11 pA/pF) compared to the control condition (black squares, n = 8; at −120 mV: −4.89 ± 0.45 pA/pF; p-value at −120 mV < 0.001). C. Normalized activation curves (I/Imax) obtained by fitting current activation to a Boltzmann function. Iso did not produce a statistically significant shift in V½ (control: −115 ± 2.7 mV; Iso: −106 ± 4.17 mV; p = 0.057) or in the slope factor (K: 24.07 ± 2.24 vs 23 ± 2.28; p = 0.8). Data are presented as mean ± SEM.
